# Supplementary material for: Hybrid Models and Biological Model Reduction with PyDSTool
Source: PLoS Comput Biol. 2012 Aug 9;8(8):e1002628. doi: 10.1371/journal.pcbi.1002628 (PMC3415397; doi:10.1371/journal.pcbi.1002628)
Supplement: Text S4 — Complete source code for the PyDSTool package (version 0.88.120504). Includes API documentation and help files linking to web pages. This file is identical to the current public release on Sourceforge.net. (ZIP) [file pcbi.1002628.s004.zip › PyDSTool/html/PyDSTool.Toolbox.optimizers.helpers.finite_difference-pysrc.html]

xml version="1.0" encoding="ascii"?


PyDSTool.Toolbox.optimizers.helpers.finite\_difference


| Home | Trees | Indices | Help | PyDSTool |
| --- | --- | --- | --- | --- |

Package PyDSTool ::
Package Toolbox ::
Package optimizers ::
Package helpers ::
Module finite\_difference

[hide private]

[frames] | no frames]

# Source Code for Module PyDSTool.Toolbox.optimizers.helpers.finite\_difference

```
  1  from __future__ import division 
  2  
 
  3  from PyDSTool import common 
  4  import numpy as np 
  5  
 


6 -class FiniteDifferencesFunction(object):


7 -    def residual(p, extra_args=None):


8          raise NotImplementedError("Define in concrete sub-class")

  9  
 


10 -class ForwardFiniteDifferences(FiniteDifferencesFunction):


11      """
 
 12      A function that will be able to computes its derivatives with a forward difference formula
 
 13      """ 


14 -    def __call__(self, params):


15          return np.linalg.norm(self.residual(params))

 16  
 


17 -    def __init__(self, eps=1e-7, *args, **kwargs):


18          """
 
 19          Creates the function :
 
 20          - eps is the amount of difference that will be used in the computations
 
 21          """ 
 22          self.eps = eps 
 23          self.inveps = 1 / eps

 24  
 


25 -    def gradient(self, params):


26          """
 
 27          Computes the gradient of the function
 
 28          """ 
 29          grad = np.empty(params.shape) 
 30          curValue = self(params) 
 31          for i in range(0, len(params)): 
 32              eps = self.eps[i] 
 33              #inveps = self.inveps[i]
 
 34              paramsb = params.copy() 
 35              paramsb[i] += eps 
 36              grad[i] = (self(paramsb) - curValue) / eps 
 37          return grad

 38  
 


39 -    def hessian(self, params):


40          """
 
 41          Computes the hessian of the function
 
 42          """ 
 43          hess = np.empty((len(params), len(params))) 
 44          curGrad = self.gradient(params) 
 45          for i in range(0, len(params)): 
 46              eps = self.eps[i] 
 47              #inveps = self.inveps[i]
 
 48              paramsb = params.copy() 
 49              paramsb[i] -= eps 
 50              hess[i] = - (self.gradient(paramsb) - curGrad) / eps 
 51          return hess

 52  
 


53 -    def hessianvect(self, params):


54          """
 
 55          Computes the hessian times a vector
 
 56          """ 
 57          raise NotImplementedError

 58  
 
 59  
 


60 -class CenteredFiniteDifferences(FiniteDifferencesFunction):


61      """
 
 62      A function that will be able to computes its derivatives with a centered difference formula
 
 63      """ 


64 -    def __init__(self, eps=1e-7, *args, **kwargs):


65          """
 
 66          Creates the function :
 
 67          - eps is the amount of difference that will be used in the computations
 
 68          """ 
 69          self.eps = eps # see the way this is used differently to inveps in gradient() below 
 70          self.inveps = 1 / (2 * eps)

 71  
 


72 -    def gradient(self, params):


73          """
 
 74          Computes the gradient of the function
 
 75          """ 
 76          grad = np.empty(params.shape) 
 77          for i in range(0, len(params)): 
 78              paramsa = params.copy() 
 79              paramsb = params.copy() 
 80              paramsa[i] -= self.eps 
 81              paramsb[i] += self.eps 
 82              grad[i] = self.inveps * (self(paramsb) - self(paramsa)) 
 83          return grad

 84  
 


85 -    def hessian(self, params):


86          """
 
 87          Computes the hessian of the function
 
 88          """ 
 89          hess = np.empty((len(params), len(params))) 
 90          for i in range(0, len(params)): 
 91              paramsa = params.copy() 
 92              paramsb = params.copy() 
 93              paramsa[i] -= self.eps 
 94              paramsb[i] += self.eps 
 95              hess[i] = self.inveps * (self.gradient(paramsb) - self.gradient(paramsa)) 
 96          return hess

 97  
 


98 -    def hessianvect(self, params):


99          """
 
100          Computes the hessian times a vector
 
101          """ 
102          raise NotImplementedError

103  
 
104  
 
105  
 


106 -class FiniteDifferencesCache(FiniteDifferencesFunction):


107      """General class for recognition by ParamEst as a function
 
108      with a non-explicit derivative. Uses a cache to save recomputation
 
109      of most recent values.
 
110      """ 


111 -    def __call__(self, params):


112          return np.linalg.norm(self.residual(params))

113  
 


114 -    def __init__(self, eps=1e-7, *args, **kwargs):


115          """
 
116          Creates the function :
 
117          - eps is the scale at which the function varies by O(1) in each
 
118          parameter direction
 
119          - grad_ratio_tol (optional, default = 10) is the relative change in any direction
 
120          after which the function is deemed to have changed non-smoothly, so that gradient in
 
121          that direction will be ignored this step
 
122          """ 
123          self.eps = eps 
124          try: 
125              self.pest = kwargs['pest'] 
126          except KeyError: 
127              # must set it using pest.setFn
 
128              self.pest = None 
129          try: 
130              self.grad_ratio_tol = kwargs['grad_ratio_tol'] 
131          except KeyError: 
132              self.grad_ratio_tol = 10

133  
 


134 -    def residual(self, p, extra_args=None):


135          # tuple(p) works for multi-parameter otherwise falls through to just p
 
136          try: 
137              pars = tuple(p) 
138          except TypeError: 
139              pars = (p,) 
140          try: 
141              r = self.pest.key_logged_residual(pars, self.pest.context.weights) 
142          except KeyError: 
143              r = self._res_fn(p, extra_args) 
144          return r

145  
 


146 -    def _res_fn(self, p, extra_args=None):


147          raise NotImplementedError("Define in concrete sub-class")

148  
 
149  
 


150 -class ForwardFiniteDifferencesCache(FiniteDifferencesCache):


151      """
 
152      A function that will be able to computes its derivatives with a
 
153      forward difference formula.
 
154      """ 


155 -    def jacobian(self, params, extra_args=None):


156          """
 
157          Computes the jacobian of the function
 
158          """ 
159          return common.diff2(self.residual, params, eps=self.eps)

160  
 


161 -    def gradient(self, params):


162          """
 
163          Computes the gradient of the function
 
164          """ 
165          # turn the matrix into a flat array
 
166          #return (common.diff2(self.__call__, params, eps=self.eps)).A1
 
167          grad = np.empty(params.shape) 
168          res_x = self.residual(params)  # will look up cache if available 
169          for i in range(0, len(params)): 
170              eps = self.eps[i] 
171              paramsa = params.copy() 
172              paramsa[i] += eps 
173              res_a = self.residual(paramsa) 
174              # filter out steps that caused non-smooth changes in gradient (as per relative change)
 
175              # i.e. make those directions count as zero
 
176              filt = (abs(res_x / res_a) < self.grad_ratio_tol).astype(int) 
177              grad[i] = (np.linalg.norm(res_a*filt)-np.linalg.norm(res_x*filt))/eps 
178          return grad

179  
 


180 -    def hessian(self, params):


181          """
 
182          Computes the hessian of the function approximated by
 
183          J^T * T
 
184          """ 
185          J = self.jacobian(params) 
186          return J.T * J

187
```

  


| Home | Trees | Indices | Help | PyDSTool |
| --- | --- | --- | --- | --- |

|  |  |
| --- | --- |
| Generated by Epydoc 3.0.1 on Fri May 4 15:24:11 2012 | http://epydoc.sourceforge.net |
